# A Case of Alveolar Bleeding from Clotting Abnormality by Cefmetazole

**DOI:** 10.1155/2019/3574064

**Published:** 2019-01-21

**Authors:** Eri Nakano, Tsubasa Fukuoka, Nao Takeuchi, Tomoyuki Seki, Michihiro Tamai, Makoto Araki

**Affiliations:** Department of Internal Medicine, Suwa Central Hospital, Chino, Nagano, Japan

## Abstract

Cephalosporins are one of the most commonly used first-line antibiotics. In this report, we describe the case of a patient who developed alveolar bleeding due to clotting abnormality following the use of cefmetazole, one of cephalosporins containing an *N*-methylthiotetrazole (NMTT) side chain. Compared to other antibiotics, cephalosporins with an NMTT side chain cause a higher degree of bleeding events. The bleeding tendency is caused by the depletion of vitamin K-dependent clotting factors via inhibition of the vitamin K epoxide reductase. This mechanism of action is the same as warfarin. Recent years have seen an increase in the number of patients using direct oral anticoagulants that do not require coagulation tests. As a consequence, there may be an increase in the number of bleeding events due to anticoagulant drugs and such antibiotics coprescription. Therefore, this case is an instructive lesson.

## 1. Introduction

Antibiotics have numerous unknown side effects. Bleeding represents one of them. The causes of antibiotics' bleeding tendency can be linked to either reduction of platelet function or deficiency of coagulation factors. Overall, the most common cause of bleeding is the deficiency of vitamin K- (Vit K-) dependent coagulation factors.

Cefmetazole (CMZ) is a second-generation cephalosporin. It is characterized by a potent antibacterial activity against Gram-negative bacteria, including *Pseudomonas aeruginosa*. CMZ is frequently used for the treatment of intraperitoneal and urinary tract infections. This cephalosporin is characterized by the presence of an *N*-methylthiotetrazole (NMTT) side chain [1].

The presence of this side chain allows CMZ to inhibit coagulation factors through the same mechanism as that of warfarin [2]. Despite causing a higher number of bleeding accidents when compared to other antibiotics, this side effect of CMZ is not well known. The present case report describes the case of a patient with pulmonary alveolar hemorrhage during treatment of urinary tract infection. We discovered that the hemorrhage was due to the inhibition of coagulation factors induced by CMZ. This case is an important example of how deficiency of Vit K-dependent coagulation factors can take place in the absence of anorexia or malabsorption when using an NMTT-group cephalosporin.

## 2. Case Report

An 84-year-old man accidentally fell at home and was admitted to our hospital. The patient was hospitalized with several problems such as multiple metastases of prostate cancer, chronic heart failure, emphysema, impaired renal function, and biliary stent placement due to idiopathic biliary stenosis. While no fracture was identified, the patient complained of lower back pain and was unable to move. As a consequence, he was hospitalized for the purpose of pain management. On the third day of hospitalization, the patient developed a fever of 38.2°C, and his laboratory data showed high levels of WBC count and CRP. While the source of infection was not identified, a urinary tract infection was suspected because he had purulent urine from previous examination and no symptom of respiratory tract infection. The patient underwent treatment with CMZ 1 g every 12 hrs. Three days after therapy initiation, the fever declined and the laboratory data of the inflammatory response normalized. Although blood culture was negative, we decided to treat according to sepsis because he was frail. We, therefore, planned to administer CMZ for 14 days. During the treatment course, the patient did not develop fever and had a healthy appetite.

**Figure 1:**
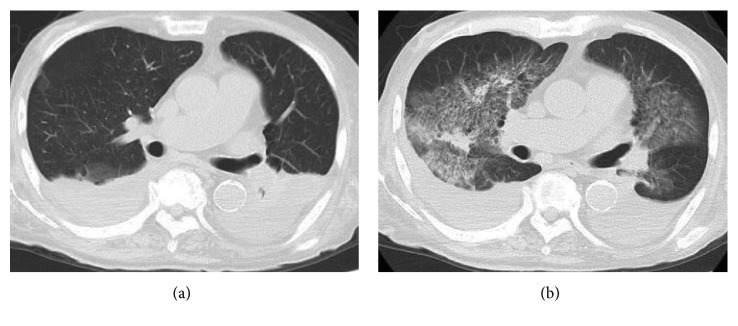
Chest CT at the time of admission (a) and 14th hospital day (b).

On the morning of the 14th day of hospitalization, the patient complained of a sudden difficulty in breathing. His peripheral artery oxygen saturation decreased to 74%. No fever, coughing, or sputum was identified. A chest computed tomography (CT) scan was performed, showing the presence of ground glass shadows bilaterally (Figure 1). While the blood work demonstrated the absence of an inflammatory response, Hb decreased by 1.5 g/dL from the previous day. The BNP value was 103 pg/dL, similar to that at initial hospitalization. Because hemostasis of the blood sampling site was difficult, additional laboratory tests were performed. These showed a marked prolongation of PT-INR (Table 1). In the evening, his value of Hb dropped from 6.8 to 5.5 g/dl in six hours. We doubted gastrointestinal bleeding, but there was no black stool. Additionally, he began to spit bloody sputum. We considered bronchoscopy but could not carry out because of his poor respiration. Since the patient had an acute respiratory failure accompanied by blood sputum and progressive anemia without exacerbation of heart failure, he was diagnosed with pulmonary alveolar hemorrhage due to coagulation abnormality. Two units of red blood cell concentrates stored in mannitol adenine phosphate and six units of fresh frozen plasma were immediately administered to the patient.

**Figure 2:**
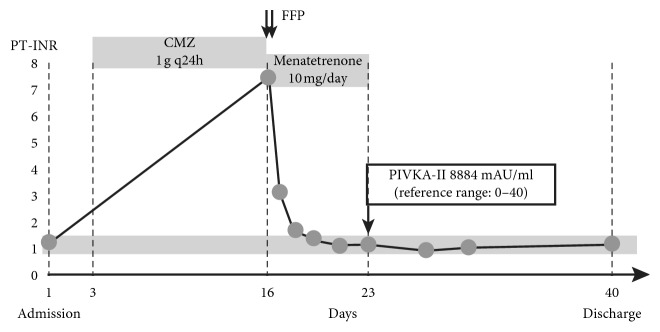
Evolution of prothrombin time values after admission.

We believe that the pulmonary alveolar hemorrhage was caused by disseminated intravascular coagulation (DIC) due to the prostate cancer. However, the patient did not meet the DIC's diagnostic criteria because each level was fibrinogen 393.8 mg/dL, fibrinogen degradation products (FDPs) 23.0 ug/dl, and platelet count 15.3 × 10^4^/uL, even though PT-INR indicated extremely abnormal value (Table 1). We took into consideration the possibility of Vit K deficiency. To overcome this issue, we administered 10 mg menatetrenone per day. Three days later, all coagulation systems had recovered to their normal values (Figure 2). Protein induced by vitamin K absence-II (PIVKAII) reached 8,884 mAU/mL (normal range below 40 mAU/mL) by the 23rd day of hospitalization.

Based on these observations, we investigated the cause behind the Vit K deficiency. The prescription drugs had not been changed before and after hospitalization, and antiplatelet and anticoagulant agents had not been used. The only additional drug used during hospitalization was CMZ. Furthermore, the patient's food intake remained unchanged in the course of the hospitalization as well as his hepatobiliary system's laboratory tests. No diarrhea developed during the patient's illness. Based on these observations, the patient was diagnosed with hypoprothrombinemia due to CMZ inhibition of Vit K epoxide reductase. Despite discontinuation of Vit K (menatetrenone) administration, the coagulation activity did not decrease. The pulmonary alveolar hemorrhage gradually improved, and the patient was discharged after one month.

One month after discharge, the patient was rehospitalized with pneumonia. He was treated with sulbactam/ampicillin 3 g quaque 12 hrs for 7 days, but hypoprothrombinemia was not observed.

## 3. Discussion

Pulmonary alveolar hemorrhage is a life-threatening condition and medical emergency. Most commonly, alveolar hemorrhage is caused by alveolar basement membrane damage due to infection, autoimmune disease, toxic exposure, or cardiac disorders [3, 4]. Although the number is small compared with those, there are numerous reports of cases in which pulmonary alveolar hemorrhage has occurred due to abnormal coagulation, following the use of warfarin [5–8] or of new generation of direct oral anticoagulants (DOACs) [9, 10].

While in the case presented here, there may have been a rise of the pulmonary artery wedge pressure due to heart failure [3, 4], and the main cause of bleeding is linked to the coagulation abnormality. Furthermore, it is very unusual that the coagulation abnormality was caused only by adverse side effects of CMZ in the absence of anorexia or malabsorption.

Bleeding tendency is a rare but an important side effect of antibiotics. There have been reports of platelet aggregation abnormalities caused by antibiotics such as carbenicillin, ticarcillin, and latamoxef [11, 12]. Coagulation abnormalities caused by antibiotics, including cephalosporin, have been reported more frequently. Coagulopathies due to antibiotics are generally caused by decreased Vit K absorption, for example, as a consequence of decreased Vit K production by intestinal bacteria following the use of antibacterial agents.

**Figure 3:**
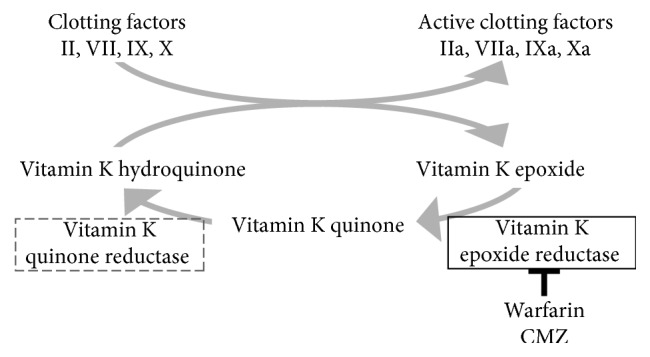
Vitamin K cycle. Warfarin and cefmetazole mainly suppresse vitamin K epoxide reductase.

Some cephalosporins with an NMTT side chain are known to cause Vit K-dependent coagulation factor deficiency by inhibiting the Vit K epoxide reductase complex subunit 1 (VKORC1) [2]. This mechanism is the same as that of warfarin (Figure 3). Therefore, the use of cephalosporins with an NMTT side chain has a higher likelihood of causing bleeding due to their action not only on intestinal bacteria but also on Vit K's cycle. Thus, the use of antibiotics with an NMTT group requires caution (Table 2).

Compared to other antibiotics, the bleeding risk linked to cephalosporins with an NMTT group was reported to be 2.9 times in CMZ and 4.6 times in CPZ/SBT [13]. Furthermore, additional risk factors linked to bleeding following antibiotics use are the use of anticoagulants, liver failure, malnutrition, and history of hemorrhagic event within 6 months [13]. The adjusted odds ratios for these risk factors are 2.08, 1.69, 1.41, and 2.57, respectively. However, none of the above-mentioned risk factors apply to this case, which instead was due to the use of CMZ. Thus, we hypothesize that the case presented here may be due to a low activity of VKORC1. It is well known that there is a wide interindividual variability in the dose of warfarin required to achieve target anticoagulation [14]. It is caused by clinical, lifestyle, or genetic factors. The VKORC1 genotypes are one of the most important known genetic determinants of warfarin dosing [15], but we have not examined in this case.

Prevention of bleeding accidents caused by cephalosporins with an NMTT group such as CMZ can be done by identifying patients at risk and monitoring coagulation following the administration of antibiotics. Special attention with careful monitoring needs to be given to patients with additional risk factors such as use of anticoagulants, liver failure, malnutrition, and/or history of hemorrhagic event within 6 months. However, even in the event of absence of risk factors, monitoring of coagulation is necessary due to the possible low activity of VKORC1 in some patients.

## 4. Conclusions

To our knowledge, this is the first case reporting pulmonary alveolar hemorrhage caused by CMZ. CMZ is frequently used due to its high antibacterial performance against *β*-lactamase-producing bacteria and its efficiency against Gram-positive and Gram-negative bacteria. However, it is not common knowledge that this drug increases the bleeding tendency at a higher degree compared to other antibiotics. As a consequence, when using cephalosporins with an NMTT group such as CMZ, it is better to perform the coagulation test at several days after the start of antibiotics therapy, just as with warfarin administration.

## Figures and Tables

**Table 1:** Laboratory findings.

	On admission	16th	40th	Unit
WBC	4270	9670	3330	/uL
Hb	8.1	6.8	9.0	g/dl
Plt	17.8	15.3	14.1	10^4^/uL
PT-INR	1.08	>7.5	1.12	
aPTT	25.7	>120	30.1	sec
Fib		393.8		mg/dl
FDP		23.0		*μ*g/dl
AT3		123		%
TP	5.4	4.6	4.7	g/dl
Alb	2.1	1.9	2.0	g/dl
AST	43	75	31	IU/L
ALT	6	6	7	IU/L
LDH	526	827	409	IU/L
T-bil	0.41	0.69	0.91	mg/dl
ALP	4300	5322	4880	IU/L
γ-GTP	48	40	101	IU/L
UA	8.2	10.5	11.0	mg/dl
BUN	29.5	25.2	33.1	mg/dl
Cr	1.21	1.22	1.09	mg/dl
CRP	1.20	4.45	2.45	mg/dl
HbA1C	5.2			%
BNP	87.1	103.0		pg/ml

FDP, fibrinogen degradation products; TP, total protein; AT3, antithrombin III.

**Table 2:** *N*-methylthiotetrazole- (NMTT-) containing antibiotics.

Antibiotics	Abbreviations
Cefmetazole	CMZ
Cefoperazone	CPZ
Cefamandole	CMD
Cefmenoxime	CMX
Cefoperazone	CPZ
Cefotetan	CTT
Cefminox	CMNX
Latamoxef	LMOX
